# Biomolecular and Biochemical Aspects of the Oral Cavity

**DOI:** 10.3390/molecules27248676

**Published:** 2022-12-08

**Authors:** Anand Marya, Dinesh Rokaya, Artak Heboyan, Gustavo Vicentis de Oliveira Fernandes

**Affiliations:** 1 Department of Orthodontics, Faculty of Dentistry, University of Puthisastra, Phnom Penh 12211, Cambodia; 2 Department of Clinical Dentistry, Walailak University International College of Dentistry, Walailak University, Bangkok 10400, Thailand; 3 Department of Prosthodontics, Faculty of Stomatology, Yerevan State Medical University after Mkhitar Heratsi, Str. Koryun 2, Yerevan 0025, Armenia; 4 Periodontics and Oral Medicine Department, University of Michigan School of Dentistry, Ann Arbor, MI 48109, USA

Recent advances in science, especially innovations in the field of biochemistry and materials science, greatly contribute to improvements in the prevention, diagnosis, and treatment of oral diseases. Biomarkers and nanoparticles found in the oral fluid may indicate physiological and pathological changes occurring not only in the oral cavity but also in other organ systems. In addition, biochemical and biomolecular changes can be induced by the use of different medications and restorative materials [1,2,3,4,5,6,7].

Liquid platelet-rich fibrin is used as a natural source of fibrinogen. Combined with artificial bone, it is used for bone augmentation in implantology. Irrespective of the relative centrifugal force, platelet-poor plasma is found to have a higher concentration of fibrinogen than that in the buffy coat fraction of liquid platelet-rich fibrin. The clottable matrix constitutes 10.2% and 25.3% of the platelet-poor plasma and buffy coat fractions, respectively. This means that the cellular components make up more than half of the coagulated buffy coat mass. The results obtained suggest that platelet-poor plasma is the primary source of clotting fibrinogen, though the buffy coat is the source of cells in sticky bone fabrication [1,8,9].

We studied the properties of different antimicrobial compounds against antibiotic-resistant bacteria and yeasts. Silver nanoparticles (AgNPs) were synthesized with the aid of wild ginger extracts. Generated in this way, AgNPs were found to have a spherical configuration, as shown by the application of scanning electron microscopy. Inexpensive, simple, and environmentally friendly, the green synthesis of AgNP is an alternative to chemical methods. Biosynthesized AgNPs exhibit a good antibacterial impact on multidrug-resistant bacterial strains. Moreover, green-synthesized AgNPs provide new opportunities for pharmaceutics to produce several pharmaceuticals, biomedical and industrial goods [2,10].

The average level of matrix metalloproteinase 9 (MMP-9) in saliva in patients with chronic periodontitis with type II diabetes was almost two times higher than in patients with chronic periodontitis only. This finding indicates that type-II diabetes mellitus (DM) influences the expression of salivary MMP-9, which can cause a deterioration in the periodontal status in type-II DM patients. Such subjects with chronic periodontitis showed considerably higher values for all the periodontal parameters, as compared to the subjects with chronic periodontitis. Periodontal status was worse in type-II DM subjects with chronic periodontitis than in non-diabetic chronic periodontitis patients [3,11]. It has been established that hyperglycemia is a key cause of increased inflammatory response, leading to a higher release of pro-inflammatory cytokines, which causes a deterioration of periodontal status.

Recent developments show that some genes are involved in signaling pathways and contribute to the progression and development of rheumatoid arthritis and periodontitis, and it has been found that these diseases are connected with each other. Moreover, IFI44Las can be used for common therapeutic purposes suppressed by vemurafenib in virtual screening and molecular docking. Yet, confirmation of these data requires the study of the cell lines [4,12]. The results of the study enhance the awareness of two inflammatory diseases and are a valuable contribution to further medication discovery research.

External apical resorptions can be conditioned by genetic factors such as interleukine-1β (IL-1β) gene, which is related to the development of inflammation induced by orthodontic treatment, and the IL-1β allele 1 has a 5.6-fold increased risk of external apical root resorption. The external apical root resorption pathogenesis and resorption-protective defensive mechanism of cement are performed by the cytokine of the IL-1β (13954) gene. The methods currently used to identify the biomarkers of root resorption are reliable and predictable. Other informative biological markers are aspartate aminotransferase, serum IgG, and salivary sIgA. Orthodontic tooth movement generates biomarkers that denote biological changes [5]. The preliminary diagnosis of external root resorption is confirmed by the cytokines and organic matrix proteins in the gingival crevice, which is released from the adjacent bone and dentin. Root resorption and periodontal pathology are characterized by an increase in the volume of gingival sulcus fluid and a change in pH. Recent studies identified 2000+ new biomarkers associated with root resorption, which allow for correct root resorption diagnosis. In addition, an increase in dentin sialoprotein and phosphoprotein were also observed, indicating physiological root resorption [5,13].

Carbopol alone was found to exhibit a poor response to mucoadhesive strength compared to agarose-based gels. It showed insufficient time for mucoadhesive flow, in vivo residence, and medicine release. Notably, the parameters improved when gels were combined. Since mucoadhesive and drug release possibilities were mostly contributed by agarose, this gel can be used to provide a mucoadhesive route for medications [6,14].

Studies show that there is 20% prevalence of sulfate-reducing bacteria in orthodontic subjects undergoing corresponding treatment with fixed appliances. Black precipitates formed in the samples are distinguishing features of iron sulfides developed from metabolism. However, more research on biochemical functions and complex interactions between sulfate-reducing bacteria are needed to confirm the cause of corrosion occurring in orthodontic materials and these can help to formulate the approaches to prevent the corrosion in the oral cavity [15,16,17].

Metformin is reported to have a direct osteogenic effect on the peri-implant tissues and lower blood sugar levels, thereby improving osseointegration. However, the establishment of the overall effect of the drug on the osseointegration of dental implants requires long-term testing in animals and humans. Thus, the routine monitoring of patients with dental implants taking metformin should be carried out. The development of dental technologies is largely made possible by various developments in the field of materials science [18,19,20]. Currently, various nanomaterials have made significant contributions to scientific innovation and technological changes in clinical dentistry. Nanoscience and nanotechnology causes in reduction in material size to the nanoscale, affecting material properties and performance, such as nano hardness, viscosity, toughness, tarnish and corrosion, creep, shrinkage, elastic modulus, strength, etc. [7,14].

We hope that the information provided in this Editorial is a major asset to further research and technological innovations in dentistry.
